# Burnout in International Medical Students: Characterization of Professionalism and Loneliness as Predictive Factors of Burnout

**DOI:** 10.3390/ijerph19031385

**Published:** 2022-01-26

**Authors:** Ivan P. Gradiski, Ana Borovecki, Marko Ćurković, Montserrat San-Martín, Roberto C. Delgado Bolton, Luis Vivanco

**Affiliations:** 1 Department of Urgent Psychiatry, University Psychiatric Hospital Vrapče, 10090 Zagreb, Croatia; 2 School of Medicine, University of Zagreb, 10000 Zagreb, Croatia; abor@mef.hr (A.B.); markocurak@gmail.com (M.Ć.); 3 Department of Statistics and Operational Research, University of Granada, 52003 Melilla, Spain; momartin@ugr.es; 4 Department of Diagnostic Imaging (Radiology) and Nuclear Medicine, University Hospital San Pedro, 26006 Logroño, Spain; rcdelgado@riojasalud.es; 5 Platform of Bioethics and Medical Education, Centre for Biomedical Research of La Rioja, 26006 Logroño, Spain; 6 National Centre of Documentation on Bioethics, Rioja Health Foundation, 26006 Logroño, Spain

**Keywords:** international medical students, burnout, empathy, lifelong learning, loneliness, family

## Abstract

Background: Burnout is a common mental problem in medical students. For those who are following medical studies abroad there is a higher risk of suffering this syndrome, due to the combination of academic stress and the stress derived from their new living situation. This study was performed with the purpose of testing the following hypothesis: in medical students enrolled in medical programs abroad, abilities associated with professionalism and family support play a protective role in the prevention of suffering burnout. Methods: A cross-sectional study was performed in the Faculty of Medicine of the University of Zagreb, where a fully English medical program is offered. The general version of the Maslach Burnout Inventory (MBI-GS) was used as a dependent variable, while Jefferson Scales of empathy, teamwork, and physician lifelong learning and the Social and Emotional Loneliness Scale for Adults were used as predictive variables. In addition, information related to sex, country of birth, native language, age, academic achievement, and living situation were collected in a socio-demographic form. Linear regression models were applied to identify predictors of burnout. Results: In a sample composed of 188 medical students (38 Croatians and 144 foreigners from 28 countries), 18% of the global score in the MBI-GS was explained by lifelong learning and family loneliness. A separate analysis for each domain of the MBI-GS allowed the creation of three models: the first model explained 19% of the variance of the “exhaustion” domain by “country of birth”, “living with parents”, “academic year”, and “cynicism”; a second model explained 24% of the variance of the “cynicism” domain by “academic year”, “empathy”, “lifelong learning”, and “exhaustion”; and finally, a third model explained 24% of the variance of the “professional efficacy” domain by “lifelong learning”, “family loneliness”, and “cynicism”. All obtained models presented an effect size between medium and large, as well as matching the required conditions for statistical inference. Conclusions: These findings confirm the important role that family plays as a source of support for medical students. Empathy and lifelong learning, two specific elements of medical professionalism, appear as protective factors in the prevention of burnout in international students.

## 1. Introduction

### 1.1. Background

Burnout is a state of mental and physical exhaustion that is frequently accompanied by the symptoms of depersonalization and low perceived accomplishment [1,2,3]. Burnout syndrome is affecting an increasing number of healthcare professionals [4,5]. This could have further burnout-related repercussions, such as diminished healthcare professional well-being, worse patient safety outcomes, and a higher incidence of medical errors [6,7,8,9,10,11,12]. While the initial focus of burnout research was on practicing physicians, increasing attention is now being paid to medical students, who are similarly affected by burnout. This type of burnout is classified as learning burnout or academic burnout, which has the same key features as job burnout, but it is limited to academic-induced burnout and only affects students [13,14]. Learning burnout is defined as a combination of emotional exhaustion, cynicism, and academic inefficacy due to their failure in meeting academic requirements [15,16].

Medical students suffer from a high rate of learning burnout, with studies estimating that every second medical student will experience burnout at some stage throughout their education [17,18]. This could have serious ramifications for their mental and physical well-being, as well as their future professional development. Learning burnout could stymie medical professionals’ advancement and have an impact on personal and professional values, such as honesty, altruism, integrity, and self-control [19,20,21]. Burnout’s negative consequences may extend outside the student years and impair the quality of health care they provide in their professional lives as practicing physicians, with issues, such as frequent medical errors, suboptimal care quality, and low patient satisfaction [20,22].

Learning burnout is affected not just by a student’s individual traits but also by the learning environment, and its onset is often triggered by academic pressure and a competitive environment [19,20,23]. Scientific research has also demonstrated that social support in the family and community can minimize the risk of burnout by increasing resilience [8]. As a result, medical students who received more social support were shown to be less likely to have burnout symptoms [24].

Because medical students studying abroad live in a new environment and are distanced from their family and friends, it could be expected that they are subjected to added stress that may be tied to an increased risk of burnout syndrome.

### 1.2. Study Purpose

Based on the aforementioned framework, students’ burnout can be defined as a general state of exhaustion in which they become cynical about the value of their studies and doubtful of their capacity to perform them correctly, similar to the previously described results in occupational groups where burnout is defined as a crisis in one’s relationship with work or the performed activity [25]. The purpose of this study was to investigate the protective role of medical professionalism, as well as social and emotional support in students who are at risk of learning burnout. With this aim, the following hypothesis was tested: personal resources associated with medical professionalism and family support have a positive impact on the prevention of burnout in medical students who are attending their professional training abroad. Four research objectives were established: (i) to measure burnout, loneliness, and specific components of medical professionalism in medical students enrolled in an international medical school at the University of Zagreb; (ii) to identify differences in the burnout measures according to sex, country of birth, the student’s specialty interest, native language, and living situation; (iii) to analyze the type of association existing between burnout and the following variables: empathy with the patients, teamwork and lifelong learning abilities, loneliness, academic achievement (represented by the academic course and the annual global score), and age; and (iv) to identify factors influencing the variability of medical students’ burnout.

## 2. Material and Methods

### 2.1. Participants

The University of Zagreb offers two medical programs: one in Croatian and one fully in English, also called international, for foreign students and Croatian students who want to obtain a professional degree in medicine in this language. In the academic year 2018–2019, when this study was performed, the complete population of undergraduate medical students attending the English medical program offered by this university was 274 students.

Inclusion/Exclusion criteria: Only undergraduate students enrolled in the English medical program were included in this study. Medical students enrolled in the Croatian medical program or attending short internship programs (i.e., Erasmus or similar) were excluded. Those students who complied with the above-mentioned inclusion criteria were invited to participate in the study.

The estimation of the sample size required for this study was calculated with the G*Power software, version 3.1.9.7. This calculation was conducted, taking into consideration the creation of three separate regression models for each domain of burnout (exhaustion, cynicism, and professional efficacy). Each regression model was based on a linear multiple regression analysis with a medium effect size (Cohen-*f*^2^ = 0.15), an alpha equivalent to 0.05, a power of 0.95, and at least 4 tested predictors from the 15 variables analyzed. Finally, 20% of questionnaires missing (questionnaires that were partially answered) was also assumed. According to this analysis, the minimum sample size should include at least 163 participants.

### 2.2. Main Measures

Burnout was measured with the General Survey of the Maslach Burnout Inventory (MBI-GS) [25]. Unlike the other versions of the MBI, the MBI-GS does not focus primarily on the service relationship but on the performance of the work in general. Since the MBI-GS was designed with the main purpose of being administered in university students and occupational groups without direct personal contact with service recipients or with only casual contact with people, it appears to be the most suitable for undergraduate medical students with limited contact with patients. The MBI-GS is composed of 16 items that are answered following a Likert scale from 0 (never) to 6 (always). The MBI-GS is composed by three domains: “exhaustion”, “cynicism”, and “professional efficacy”. The exhaustion domain includes references to both emotional and physical fatigue but does not make direct reference to people as the source of those feelings. The cynicism domain reflects indifference or a distant attitude toward work (for example, “I don’t really care if my work is done well or poorly”) not to interpersonal relationships at work. Finally, the professional efficacy domain encompasses both social and non-social aspects of occupational accomplishments. This domain explicitly assesses an individual’s expectations of continued effectiveness at work. Together the three domains of the MBI-GS provide a three-dimensional measure of burnout. A high degree of burnout is reflected in high scores on “exhaustion” and “cynicism” and low scores on the “professional efficacy” domain.

Empathy was measured with the medicine student version of the Jefferson Scale of Empathy (JSE-S) [26]. The JSE-S evaluates students’ orientation toward empathetic relationships with patients. The scale is composed by 20 items that are answered following a Likert scale from 1 (strongly disagree) to 7 (strongly agree). A higher score means that the student has a greater orientation or behavioral tendency towards empathic engagement in patient care.

Teamwork abilities, also called abilities towards inter-professional collaborative work between nursing and medicine, were measured with the Jefferson Scale of Attitudes toward Physician-Nurse Collaboration (JSAPNC). The JSAPNC is a valid instrument to measure teamwork and inter-professional collaboration abilities between physicians and nurses [27]. The JSAPNC responds to the definition of teamwork as an ability of nurses and physicians to work together cooperatively, sharing responsibilities for solving problems and making decisions to formulate and carry out plans for patient care. The 15 items of the JSAPNC are answered using a Likert scale from 1 (strongly disagree) to 4 (strongly agree).

Lifelong learning was measured with the medicine student version of the Jefferson Scale of Physicians Lifelong Learning (JeffSPLL-MS) [28]. The JeffSPLL-MS measures the development of skills related to information gathering, the use of learning opportunities, and self-motivation. The scale is composed of 14 items that are answered following a Likert scale from 1 (strongly disagree) to 4 (strongly agree).

Loneliness was measured using the short version of the Social and Emotional Loneliness Scale for Adults (SELSA-S) [29]. The SELSA-S is a multidimensional measure of loneliness, following the conceptualization initially proposed by Weiss [30]. The SELSA-S is composed of three domains: social, family, and romantic loneliness. The social loneliness domain indicates a lack of an engaging social network (related to friendships and workplace relationships). The family loneliness domain reflects deficiencies in close attachment in caregiver-child relationships (lack of guidance, advice, and support in the family environment). Finally, the romantic loneliness domain indicates a lack of attachment in intimate romantic relationships (deficiencies in dating frequency, romantic involvement, and dyadic adjustment in intimate relationships). The following are sample items from each of the SELSA-S domains: “I do not feel satisfied with the friends that I have” (social loneliness), “I feel alone when I’m with my family” (family loneliness), and “I have a romantic partner to whose happiness I contribute” (romantic loneliness). The SELSA-S produces a global loneliness score based on 15 items, as well as scores for its 3 domains. Higher scores in the scale indicate a higher perception of loneliness. The SELSA-S is composed of 15 items that are answered following a Likert scale from 1 (strongly disagree) to 7 (strongly agree).

Finally, sex, age, country of birth (Croatia or other), living situation (living with parents or not), enrolled academic course, native language (native English speaker or not), and the student’s specialty interest were collected in a complementary form.

### 2.3. Procedures

The aforementioned measures were included in a unique questionnaire. This questionnaire was administered to undergraduate medicine students enrolled in a fully English medical program at the Faculty of Medicine of the University of Zagreb, in Croatia. The questionnaires consisted of paper forms provided together with a pencil and an information letter in enclosed envelopes. Once questionnaires were completed, they were returned in their envelopes to local researchers, following a general protocol previously approved by an independent ethics committee (Ref. 380-59-10106-17-100/159). The University of Zagreb provided administrative support for the process of distribution and collection of the questionnaires. The participation of medical students was voluntary, anonymous, and secret. The process was carried out in accordance with the ethical principles for medical research involving human subjects of the Declaration of Helsinki. There was no potential risk for participants, and anonymity was guaranteed throughout the entire data collection and subsequent analysis.

### 2.4. Data Analysis

Only the scales with fully completed scores in all their items were included into the statistical analysis. The reliability of each scale used was assessed by calculating Cronbach’s alpha coefficient. The scales were considered satisfactory when values were higher than 0.70. Only the global score of the MBI-GS followed a normal distribution. A normal distribution was not achieved in the remaining scales, in the sub-scales for the three domains of burnout, or in numerical variables collected. Therefore, in comparative analyses by categorical variables the parametric tests (t-test and ANOVA) were used only in the case of global scores of burnout, while nonparametric tests (Mann–Whitney U and Kruskal–Wallis tests) were used in the case of the “exhaustion”, “cynicism”, and “professional efficacy” domains. Spearman correlation coefficients were used to estimate the existence of an association between the burnout measures and the scales of empathy, teamwork, lifelong learning, and loneliness. Correlation analyses, using Spearman correlation coefficients, were also used to assess the association between burnout and academic course, annual global scores, and age.

A multiple linear regression analysis was performed using burnout measures as dependent variables, and all variables that showed statistical significance in the previous analyses were treated as possible explanatory variables. Thanks to these analyses, it was possible to create models that enabled the identification of variables acting as predictors for the global score of burnout and for the scores of the “exhaustion”, “cynicism” and “professional efficacy” domains. In all cases, the obtained models met the necessary conditions for statistical inference. That is, normality, zero mean, constant variance, and independence of the residuals, in addition to linearity and the absence of multi-collinearity. Finally, in order to quantify the degree of practical significance of the findings observed in each model, the effect size (Cohen’s *f*^2^) was calculated. An effect size equal to 0.02 was interpreted as small, equal to 0.15 was interpreted as medium, and equal to 0.35 was interpreted as large [31].

All analyses were performed in R language, version 3.6.2 for Windows. In the statistical analysis the nortest [32], apaTables [33], rstatix [34], and multilevel [35] packages were used.

## 3. Results

The entire sample included 188 medical students (88 male and 100 women), which represented 77% of all medical students enrolled in this program. Of them, 38 were Croatians, while the other 144 belonged to 28 countries. The majority of the foreigner student group were original from Europe (64 students from 16 countries) and Israel (54 students). The remaining 26 students were born in eleven countries distributed in North America, Asia, and Africa. From the entire sample, only 22 students were native English speakers. By age, the average was 23 years old (*SD* = 3), with a range between 18 and 41 years old. According to their living situation, 61 respondents were living with their parents. According to the academic achievements, 55 students were from the first course, 42 from the second, 27 from the third, 22 from the fourth, 20 from the fifth, and 21 from the sixth course. Regarding students’ medical interest, 57 students indicated that they still had not decided what to choose, while the other 131 students were distributed among 19 specialties, as follows: 50 students chose surgical specialties, 24 students chose primary care or mainly people-oriented specialties (psychiatry, family medicine, and pediatrics), and 55 students chose specialties not included in these two groups.

The first research objective was the measurement of burnout, loneliness, and the three components of medical professionalism (empathy, teamwork, and lifelong learning). All scales showed reliability, with Cronbach’s alpha coefficients higher than 0.70. A summary of the distribution of the scores achieved for each scale is shown in Table 1.

Regarding the second research objective, no statistically significant differences appeared in the measurement of burnout by sex, country of birth, the student’s specialty interest, native language, and living situation in the global score or in the separate domains.

Correlation analyses (third research objective) confirmed the existence of an inverse association between the global scores of burnout and medical professionalism for empathy (ρ = −0.27; *p* < 0.001) and for lifelong learning (ρ = −0.36; *p* < 0.001) but not for teamwork abilities (ρ = −0.09; *p* = 0.22). On the other hand, a direct correlation appeared between the global perception of burnout and family (ρ = +0.31; *p* < 0.001) and social loneliness (ρ = +0.28; *p* < 0.001) but not between burnout and romantic loneliness (ρ = −0.04; *p* = 0.64). Finally, annual course score (ρ = −0.06; *p* = 0.55), academic courses (ρ = −0.06; *p* = 0.44), and age (ρ = −0.06; *p* = 0.41) did not show an association with the global perception of burnout. The complete summary of this analysis by each domain of the MBI-GS is reported in Table 2.

**Figure 1:**
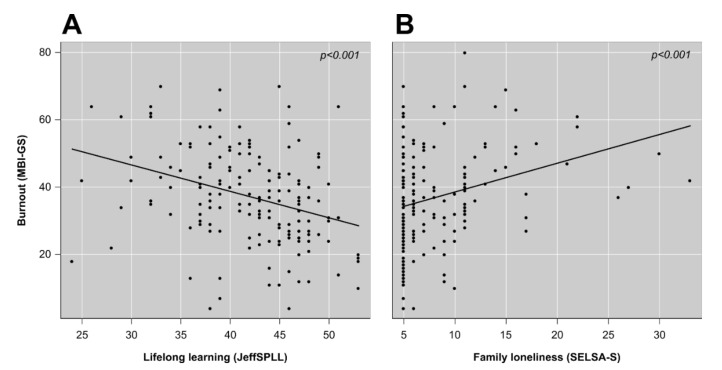
Variation in the global scores of burnout as a function of lifelong learning (**A**) and family loneliness (**B**).

**Figure 2:**
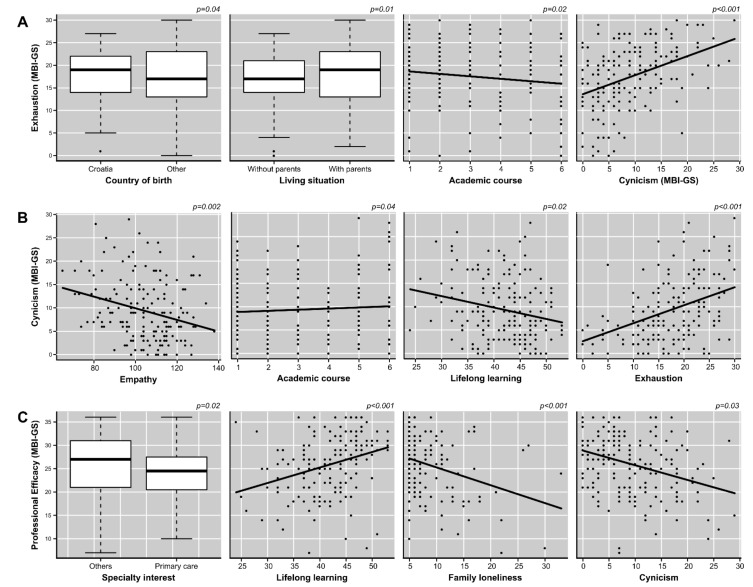
Factors influencing the variation of the scores of the exhaustion (**A**), cynicism (**B**), and professional efficacy (**C**) domains.

The fourth research objective was to identify which variables had a role of influence in the measures of students’ burnout, in order to test the hypothesis of this study. A multiple linear regression analysis allowed the creation of a model explaining 18% of the variability of the global score of the MBI-GS (R^2^-adjusted = 0.17; *F*_(2,162)_ = 17.75; *p* < 0.001; Cohen’s *f*^2^ = 0.22) based on the following variables: “lifelong learning”, with a negative linear relationship, and “family loneliness”, with a positive linear relationship (Figure 1). In addition, separate linear regression analyses were performed to determine influencing factors for each domain of the MBI-GS. These analyses allowed the creation of three models: The first model explained 19% of the variability of the “exhaustion” domain (R^2^-adjusted = 0.17; *F*_(4,162)_ = 9.62; *p* < 0.001; Cohen’s *f*^2^ = 0.24). This model included the variables of “country of birth (other different than Croatia)” and “academic year”, with negative linear relationships, and “living situation (living with their parents)” and “cynicism”, with a positive linear relationship (Figure 2A). The second model explained 24% of the variability of the “cynicism” domain (R^2^-adjusted = 0.22; F_(4,163)_ = 13.04; *p* < 0.001; Cohen’s *f*^2^ = 0.32). This model included the variables of “empathy” and “lifelong learning”, with negative linear relationships, and “academic year” and “exhaustion”, with positive linear relationships (Figure 2B). Finally, the third model explained 24% of the variability of the “professional efficacy” domain (R^2^-adjusted = 0.22; *F*_(4,159)_ = 12.31; *p* < 0.001; Cohen’s *f*^2^ = 0.31). This model included the variables of “lifelong learning”, with a positive linear relationship, and “specialty interest (primary care)”, “family loneliness”, and “cynicism”, with negative linear relationships (Figure 2C). All models presented a size effect between medium and large and matched the required conditions for statistical inference. The variance inflation factors for all assessed predictors were below 1.5. The complete summary of these analyses is shown in Table 3.

## 4. Discussion

The main aim of this study was to investigate the potential protective role that medical professionalism and family support have in the prevention of burnout in students enrolled in international medical programs. Based on this premise, the target group of this study were students enrolled in the English medical program carried out by the Faculty of Medicine of the University of Zagreb in Croatia. The participants included in this study accounted for three-quarters of all medical students enrolled in this program. Following findings reported in previous studies [36,37], the authors predicted that the majority of overseas students would be experiencing separation from their loved ones, with the added stress of training in a foreign environment. On the other hand, it was expected that Croatian students, who were also part of the study sample, experienced some level of stress derived from attending their medical training in a language different than Croatian.

### 4.1. Positive Correlation between Burnout and the Measures of Loneliness and Medical Professionalism

Findings observed in this study confirmed a positive correlation between burnout and family and social loneliness, but not romantic loneliness. These findings were somewhat expected by the authors, taking into consideration that international students were living and studying separated from their families and friends. These findings are also in consonance with studies previously reported in other cultural environments. Gil-Calderon and colleagues reported in Spanish medical students that family support is linked to lower scores on burnout measures [38]. In the United States, a positive family environment has been described as a source of happiness and a pleasant home atmosphere that allows medical students to relax from rigorous study, evaluations, and stressful clinical scenarios [39]. In China, Chunming and colleagues [18] reported a positive correlation between burnout syndrome and social isolation in medical students. They also found that having a close family relationship is protective against burnout in Chinese medical students [18]. However, the abovementioned correlation was not confirmed in the case of family loneliness and burnout. It does not necessarily mean that romantic relationships are not important. In fact, a recently published review highlighted that married medical students with strong social connections with their families have a lower perception of stress in comparison with those who are single [40]. In previous studies with healthcare professionals from Chilean [41] and Spanish [42] institutions, authors also reported a positive correlation between romantic loneliness and burnout and self-perceived measures of somatization, exhaustion and work alienation. Therefore, it is more plausible that the lack of correlation can be explained by the mean age and the living situation of the study group. Furthermore, in European society, similar as in other western societies, there is certain social tendency to postpone partner relationships until professional studies are finished or after economic independency is reached.

Regarding the association between the measured components of medical professionalism and burnout, analyses confirmed a negative correlation between global scores of burnout and global scores of empathy and lifelong learning but not teamwork. These findings are in consonance with other previously reported studies, where burnout and empathetic abilities [19,41,43] and learning environments [44] were assessed.

### 4.2. Characterization of Predictors for Global Burnout

Aware of the difficulty of establishing immediate cause-effect relationships in observational studies, linear regression analyses were performed to determine the role that family environment, medical professionalism and other individual characteristics play in students’ burnout. Specifically, in the case of the global perception of burnout, family loneliness and lifelong learning appeared as positive and negative predictors of burnout, respectively. These findings are in consonance with others reporting a protective role of learning environments and learning abilities in the prevention of academic burnout in medical students [44]. In the case of family loneliness, these findings bring new evidence supporting the negative effect that it has on medical students’ well-being. In a recent study performed in Peru, authors reported the negative impact that family loneliness has in the early development of professional competences in medical students [45]. The findings reported in this study highlight the importance that family environment has for overseas medical students and for students enrolled in medical programs in a foreign language.

### 4.3. Characterization of Predictors for Each Domain of Burnout

In order to have a deeper understanding of those elements that are influencing the perception of burnout, a separate linear regression analysis was performed to characterize predictors for each of its three domains: “cynicism”, “exhaustion”, and “professional efficacy”.

In the first case, empathy and lifelong learning appeared as protective factors against cynicism, while academic year appeared as a risk factor. Taking into account that medical empathy is predominantly cognitive (rather than emotional), this finding suggests that educative interventions aimed at strengthening empathetic abilities in medical students could contribute to the reduction in students’ cynicism. In relation with lifelong learning, it is well established that medical students suffering burnout tend to have a poor academic performance [46]. The findings observed in this study suggest that an enhancement of lifelong learning abilities could have a positive impact on the prevention of burnout derived from the reduction in cynicism. The model obtained also showed that cynicism is higher in medical students enrolled in advanced courses. This is consistent with a longitudinal study recently published by Kachel and colleagues in Austria [47], which reported that medical students showed a gradual development of cynicism in time. They stated that the phenomenon observed in the Austrian medical students was caused by the medical training process and the learning environment (formal, informal, and hidden curriculum), which is well documented in the literature [48]. Therefore, the authors believe that interventions focused on the reduction in cynicism that are based on the enhancement of empathy and lifelong learning abilities could be more effective if they are performed in the earliest stages of medical training. In fact, in the case of empathy, its enhancement in the early stages of medical training has been demonstrated in two recently published interventional studies performed with medical students from the Dominican Republic [49] and Peru [50]. The early enhancement in other competencies, such as teamwork, has also been shown to be beneficial for the improvement of empathy and lifelong learning abilities, as authors have demonstrated in another study carried out with medical and nursing students in Mexico [51].

In the case of exhaustion, living with parents was found to be the strongest predictor, while being an international student with a country of birth other than Croatia and being in a more senior year of medical studies were revealed to be protective factors. Initially, it was expected that international students could show higher levels of stress than those who were studying in their homeland. However, these results have shown that living with parents adds to a student’s exhaustion, while living separately from them is linked to lower levels of exhaustion. This finding is consistent with those reported by Shadid and colleagues. They stated that stress is more prevalent in students who live with their parents or relatives in comparison with those who do not [52]. However, according to another study performed in different European institutions, freshman students who live with their families experience less burnout and stress than those who live away from home [53]. According to this, while living with parents contributes to exhaustion, due to additional familial responsibilities and adherence to familial rules, the increased familial support outweighs the stress that accompanies the process of adaptation in newly enrolled students. However, studying medicine usually implies an important investment for families, due to the effort, time, and dedication that it requires in comparison with other disciplines. Therefore, it is reasonable that medical students living with their parents perceive more commitment towards their caregivers in time. Consequently, it is possible that those students are under more stress derived from seeing themselves more often as a family burden. Something that is probably less frequent in those who are not living with their families and handling their daily difficulties more privately. This phenomenon has been recent demonstrated in one study performed with Peruvian medical students living in different family environments [45]. Perhaps, the ones benefiting most can be those students for whom living separate from their parents is not a barrier for receiving adequate support and attachment to their family. However, additional research could provide valuable insight into this finding. The regression model also showed that, different from cynicism, attending an advance course contributes to a reduction in exhaustion. The acquisition of personal resources to overcome challenges, such as language and cultural barriers, in more experienced medical students could be explained by this difference.

According to the obtained model for professional efficacy, a specialty interest in primary care and family loneliness have a negative impact on this component, while lifelong learning shows a positive influence on its development. Previous studies have demonstrated a greater development of lifelong learning abilities in physicians coming from surgical and technical specialties and in those who have more dedication to academic activities [54]. In addition, research demonstrates that medical students and young physicians interested in people-oriented medical branches (such as primary care, pediatrics, or psychiatry) tend to have a greater development in abilities oriented to their patients’ needs compared to those that have a direct benefit on themselves. This is the case of abilities related to teamwork [55] and empathy [56] but not lifelong learning. The negative impact of family loneliness on the development of professional efficacy is also in consonance with the important role of support that the family environment plays for students’ academic achievements, as previously described.

### 4.4. Limitiations of the Study

The study included a heterogeneous group of students from different geographical and cultural backgrounds. In addition, all questionnaires used were administered in English, which was not the native tongue of the majority of the students who composed the study sample. However, taking into account that one of the requirements for the enrollment in the International medical program at University of Zagreb is a proficient knowledge of English, the authors considered that this limitation was not crucial.

Another important aspect is related to the fact that this study was conducted at a single institution, and its generalizability is, therefore, limited. In addition, the findings reported in this study explain only a small part of the variability of burnout that international medical students perceive. It is evident that it is a complex phenomenon where more aspects should be taken into consideration, such as those related to personality traits, cultural, social, and family backgrounds and others not necessarily related to the academic environment. All those aspects probably play an important role of influence in a more effective integration of these students into their new academic environment. However, the evidence reported in this study could help medical educators design curricular and extra-curricular interventions to improve student well-being and professional performance.

## 5. Conclusions

The findings of this study provide new evidence to help reach a better understanding of the international students’ acculturative stress associated with separation from their family support. The evidence reported in this study suggests that this phenomenon should not be overlooked. Institutional resources should be directed toward meeting these needs in order to maintain international students’ health and professional development during their academic period. Interventions focused on fostering attributes and skills, including specific components of medical professionalism, such as lifelong learning and empathic abilities, and social support, could be crucial for minimizing and preventing students’ burnout. The aforementioned interventions, suggested based on these findings, could have an important role in the improvement of the academic performance of students enrolled in a foreign language medical program and in overseas medical students. Based on the aforementioned findings, interventional strategies could be more effective if they are performed in the first stages of the medical training.

## Figures and Tables

**Table 1:** Descriptive analysis of the measures of burnout, loneliness, empathy, teamwork, and lifelong learning.

Scales	*n*	PR	AR	M (*SD*)	Mdn	Reliability
MBI-GS	171	0–96	4–80	37 (15)	36	0.83
Exhaustion	179	0–30	0–30	18 (7)	18	0.84
Cynicism	175	0–30	0–29	9 (7)	8	0.75
Professional Efficacy	173	0–36	0–36	26 (6)	27	0.77
SELSA-S	178	15–105	15–86	37 (14)	37	0.82
Family domain	186	5–35	5–33	8 (5)	6	0.84
Romantic domain	179	5–35	5–27	18 (10)	21	0.89
Social domain	187	5–35	5–29	11 (5)	9	0.84
JSE-S	182	20–140	65–138	105 (14)	106	0.82
JSAPNC	181	15–60	29–59	47 (6)	47	0.81
JeffSPLL-MS	176	14–56	24–53	42 (6)	42	0.76

MBI-GS, General Survey of the Maslach Burnout Inventory; SELSA-S, Family Domain of the Social and Emotional Loneliness Scale for Adults; JSE, Jefferson Scale of Empathy; JSPANC, Jefferson Scale of Attitudes toward Physician-Nurse Collaboration; JeffSPLL, Jefferson Scale of Physicians Lifelong Learning.

**Table 2:** Spearman correlation coefficients for the Maslach Burnout Inventory scores according to loneliness, empathy, teamwork, lifelong learning, academic achievement, and age.

	MBI-GS			
	Global Score	Exhaustion	Cynicism	Professional Efficacy
*Medical professionalism*				
Empathy	−0.27 ***	−0.10	−0.28 ***	+0.17 *
Teamwork	−0.10	−0.01	−0.20 **	−0.05
Lifelong learning	−0.36 ***	−0.16 *	−0.24 **	+0.39 ***
*Loneliness*				
Global score	+0.14	+0.07	+0.08	−0.12
Family loneliness	+0.31 ***	+0.20 **	+0.20 **	−0.23 **
Romantic loneliness	−0.04	−0.02	−0.08	−0.02
Social loneliness	+0.28 ***	+0.22 **	+0.19 *	−0.16 *
*Academic achievement*				
Global course score	−0.06	−0.10	−0.13	−0.03
Course of studies	−0.06	−0.13	+0.003	−0.06
*Age*	−0.07	−0.09	−0.07	+0.01

* *p* < 0.05; ** *p* < 0.01; *** *p* < 0.001.

**Table 3:** Multiple linear regression models for the Maslach Burnout Inventory (MBI-GS) and its domains.

Predictors	*β*	*SE*	*t*	*p*	VIF
*Global burnout*					
Lifelong learning	−0.74	0.17	−4.43	<0.001	1.00
Family loneliness	+0.77	0.20	+3.87	<0.001	1.00
*Exhaustion domain*					
Cynicism	+0.41	0.07	+5.49	<0.001	1.01
Country of birth (different than Croatia)	−2.72	1.37	−1.99	0.04	1.41
Living with parents (yes)	+2.92	1.18	+2.48	0.01	1.35
Academic year	−0.66	0.29	−2.27	0.02	1.08
*Cynicism domain*					
Exhaustion	+0.34	0.07	+5.22	<0.001	1.04
Empathy	−0.10	0.03	−3.16	0.002	1.02
Lifelong learning	−0.17	0.07	−2.36	0.02	1.05
Academic year	+0.51	0.26	+1.98	0.04	1.03
*Professional efficacy*					
Lifelong learning	+0.28	0.07	+3.74	<0.001	1.06
Family loneliness	−0.35	0.09	−3.89	<0.001	1.08
Speciality (Primary care)	−3.35	1.38	−2.43	0.02	1.03
Cynicism	−0.16	0.07	−2.22	0.03	1.11

*β*, beta coefficient; *SE*, standard error; *t*, t experimental; *p*, *p*-value; *VIF,* variance inflation factor.

## Data Availability

Data are available upon request.
